# Clinical, Neuroimaging and Robotic Measures Predict Long-Term Proprioceptive Impairments following Stroke

**DOI:** 10.3390/brainsci13060953

**Published:** 2023-06-15

**Authors:** Matthew J. Chilvers, Deepthi Rajashekar, Trevor A. Low, Stephen H. Scott, Sean P. Dukelow

**Affiliations:** 1 Department of Clinical Neurosciences, Cumming School of Medicine, University of Calgary, 3330 Hospital Drive NW, Calgary, AB T2N 4N1, Canada; matthew.chilvers@ucalgary.ca (M.J.C.); deepthi.rajasheka1@ucalgary.ca (D.R.); talow@ucalgary.ca (T.A.L.); 2 Hotchkiss Brain Institute, University of Calgary, 3330 Hospital Drive NW, Calgary, AB T2N 4N1, Canada; 3 Department of Biomedical and Molecular Sciences, Queens University, Kingston, ON K7L 3N6, Canada; steve.scott@queensu.ca; 4 Centre for Neuroscience Studies, Queens University, Kingston, ON K7L 3N6, Canada; 5 Providence Care Hospital, Kingston, ON K7L 3N6, Canada

**Keywords:** proprioception, stroke, prediction, robotics, modeling, stroke recovery, stroke outcomes

## Abstract

Proprioceptive impairments occur in ~50% of stroke survivors, with 20–40% still impaired six months post-stroke. Early identification of those likely to have persistent impairments is key to personalizing rehabilitation strategies and reducing long-term proprioceptive impairments. In this study, clinical, neuroimaging and robotic measures were used to predict proprioceptive impairments at six months post-stroke on a robotic assessment of proprioception. Clinical assessments, neuroimaging, and a robotic arm position matching (APM) task were performed for 133 stroke participants two weeks post-stroke (12.4 ± 8.4 days). The APM task was also performed six months post-stroke (191.2 ± 18.0 days). Robotics allow more precise measurements of proprioception than clinical assessments. Consequently, an overall APM Task Score was used as ground truth to classify proprioceptive impairments at six months post-stroke. Other APM performance parameters from the two-week assessment were used as predictive features. Clinical assessments included the Thumb Localisation Test (TLT), Behavioural Inattention Test (BIT), Functional Independence Measure (FIM) and demographic information (age, sex and affected arm). Logistic regression classifiers were trained to predict proprioceptive impairments at six months post-stroke using data collected two weeks post-stroke. Models containing robotic features, either alone or in conjunction with clinical and neuroimaging features, had a greater area under the curve (AUC) and lower Akaike Information Criterion (AIC) than models which only contained clinical or neuroimaging features. All models performed similarly with regard to accuracy and F1-score (>70% accuracy). Robotic features were also among the most important when all features were combined into a single model. Predicting long-term proprioceptive impairments, using data collected as early as two weeks post-stroke, is feasible. Identifying those at risk of long-term impairments is an important step towards improving proprioceptive rehabilitation after a stroke.

## 1. Introduction

### 1.1. Proprioception and Its Importance after Stroke

Proprioception, described by Sir Charles Sherrington [1], refers to the sense of limb position and movement, originating from receptors within the muscles and joints themselves [2]. The proprioceptive sense is important in allowing us to move our limbs freely in space and interact with our surroundings. Following stroke, proprioceptive impairments are common, typically observed in approximately 50% of stroke survivors [3,4]. Proprioceptive impairments have been associated with a reduced ability to perform activities of daily living (ADLs) post-stroke, independent of motor deficits [5,6,7,8]. Mitigating these impairments can be important for restoring independent quality of life. Recent studies assessing proprioception using robotic technology have, however, demonstrated that impairments are still apparent in 20–40% of stroke survivors six-months after stroke [9,10]. This is not necessarily surprising considering that evidence-based interventions for treating proprioception post-stroke are lacking (for an in-depth review, see [11,12]). Unfortunately, little is often done clinically to target proprioceptive recovery, and more than half of therapists believe that current treatments for somatosensory impairment are ineffective, lacking confidence in their ability to treat somatosensory deficits [13]. Early identification of individuals at high-risk of suffering chronic proprioceptive impairments is an important step towards personalized approaches to rehabilitation. Doing so would highlight those who would benefit from rehabilitation with some focus on restoring proprioception.

### 1.2. Predictors of Proprioceptive Impairment 

Within stroke centres, clinical assessments and neuroimaging are a standard component of patient care, and thus are readily available for use in predicting patient outcomes. Previous literature has assessed the relationship of many clinical and neuroimaging measures with assessments of proprioception in the subacute stage post-stroke [7,14,15,16,17,18]. In particular, clinical measures of attention and activities of daily living, such as the Behavioural Inattention Test (BIT) and Functional Independence Measure (FIM), have been closely associated with measures of proprioception [7,14,15,16,17,18]. Furthermore, studies have investigated the relationship between neuroimaging measures, such as lesion volume and region-specific damage, and robotic assessments of proprioception [10,16,19,20,21,22]. Greater lesion volume has been linked with a worse proprioceptive performance post-stroke [16,19,20,21], while additional studies have used voxel-based lesion-symptom mapping (VLSM) to assess the statistical relationships between lesioned brain regions and proprioceptive performance after stroke [10,20,21,22]. In comparison to motor recovery, where many studies have tried to identify early predictors of recovery [23,24,25,26,27,28], few have focused on predicting long-term proprioceptive recovery [10,19,29].

### 1.3. Aims and Hypothesis

The utility of clinical, neuroimaging and robotic measures in predicting and classifying long-term proprioceptive outcomes has been under-explored. The purpose of this study, therefore, was to evaluate the ability of clinical, neuroimaging and robotic measures, collected within the first two weeks post-stroke, to predict proprioceptive impairments six-months post-stroke both independently of and in combination with each other. Considering the previously established associations between clinical measures, neuroimaging and proprioceptive measures, it was hypothesized that models containing just clinical or just neuroimaging features would reasonably predict six-month proprioceptive impairments. Furthermore, it was hypothesized that robotic features, alone and in conjunction with clinical and neuroimaging features, would lead to superior predictions in terms of accuracy and area under the receiver-operator characteristic (ROC) curve (AUC). 

## 2. Materials and Methods

### 2.1. Participant Recruitment

Participants for the current study were recruited from a pool of participants taking part in a larger, ongoing prospective cohort study called RESTART, which documents stroke recovery using robotics and neuroimaging over the first six-month post-stroke. 

#### 2.1.1. Study Inclusion Criteria

Participant inclusion criteria for the current study was: (1) 18+ years of age, (2) first time ischemic or hemorrhagic unilateral stroke, (3) could follow task instructions, (4) completed a robotic arm position matching (APM) task at both two weeks and six months post-stroke, (5) had a clinical assessment collected at approximately two weeks post-stroke and (6) had clinical neuroimaging collected (Magnetic Resonance Imaging (MRI) or Computed Tomography (CT)).

#### 2.1.2. Study Exclusion Criteria

Participant exclusion criteria were: (1) apraxia [30], (2) contraindications to neuroimaging, (3) other diagnosed neurological disorders (multiple sclerosis, Parkinson’s disease, etc.), or (4) upper extremity orthopedic injury/pain that impacted their ability to perform the robotic assessments.

### 2.2. Robotic Assessment of Proprioception

**Figure 1:**
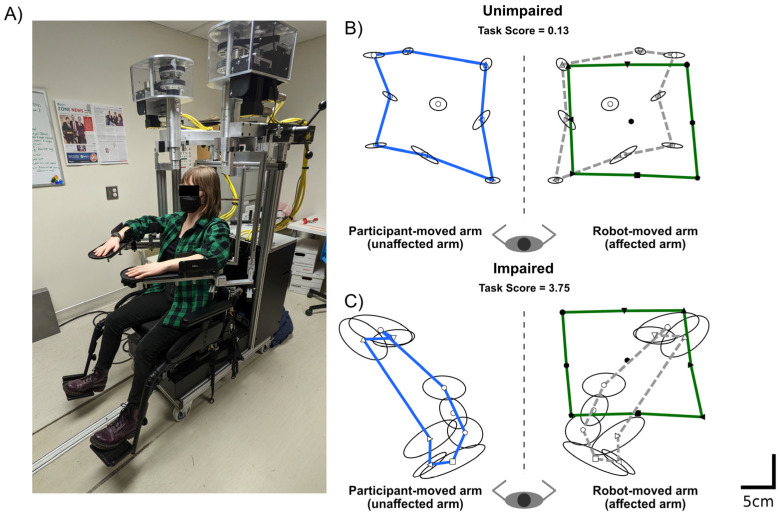
Robotic Assessment of Proprioception—(**A**) Kinarm Exoskeleton Lab (Kingston, ON, Canada) used to perform the robotic assessment of proprioception (Arm Position Matching task). (**B**) Exemplar Arm Position Matching task performance of an unimpaired stroke participant. The robot moved the affected right arm. Closed symbols indicate the mean nine target positions where the robot moved the participant’s hand. For illustrative purposes, the green line connects the mean hand position of the eight outer targets. The participant matched with their unaffected left arm. Open symbols indicate the mean, matched hand position of the unaffected arm. Again, for illustrative purposes, the blue line connects the eight outer targets. Ellipsoids represent the variability (one standard deviation) in matched position around each target. For illustrative purposes, the participant’s matched positions have been reflected across the midline onto the robot moved arm. The dashed grey line connects the reflected outer 8 targets. (**C**) Same format as B, except an exemplar from a stroke participant with an impairment on the Arm Position Matching task is provided.

Proprioception was assessed in the current study, using a robotic APM task [3,7], at two time points post-stroke, approximately two weeks (12.4 ± 8.4 days) and six months (191.2 ± 18.0 days) post-stroke. The APM task was performed in a Kinarm Exoskeleton robotic device (Kinarm., Kingston, ON, Canada). Participants sat in the wheelchair base of the robotic device with their arms supported in the horizontal plane by custom-fitted arm-troughs (Figure 1A). The linkages on the robot were then adjusted to fit each participant, such that the length of the robotic arms matched those of the participant and the robotic joints lined up with the participant’s shoulders and elbows. Once each participant was set up, they were wheeled into the virtual reality environment, and vision of the arms was occluded by an opaque shutter and bib. 

The APM task began with the robot moving the participant’s affected arm to one of nine-spatial targets. These targets were oriented in a 20 cm × 20 cm square, with eight outer targets surrounding a central target. Once the robot had finished moving the participant’s affected arm to the first target, they were instructed to attempt to mirror match the position of the robot-moved (affected) arm, with their unaffected arm. Participants then verbalized that they felt that they were in a matched position, and the robot operator cued the robot to move the arm to the next target. The robot moved the participant’s arm between the nine targets with a bell-shaped velocity profile and a maximum speed of 0.3–0.5 m/s. Each target was assessed in a pseudorandomized order, once per block. Six blocks were performed, so that there were 54 trials in the APM task. Exemplars of the APM task, from participants with and without an APM task impairment, are shown in Figure 1B,C, respectively.

Robotic assessments allow for objective measures of sensorimotor function, compared to standard clinical assessments [31]. As such, performance on the APM task was used as the primary measure of proprioception, to classify participants to those with and without impairments at six months post-stroke. Performance parameters from the APM task are described in Section 2.3. The APM task parameters used as features in the predictive models are described in Section 2.6.

### 2.3. Quantifying Proprioceptive Performance and Impairments

Performance on the APM task was quantified by a global measure called an APM Task Score, which was used to infer the presence of proprioceptive impairments. The APM Task Score is a composite measure, based on a number of parameters, each reflective of a different component of APM task performance. These parameters include: (1) Absolute Error, which quantifies the absolute distance in the mirror-matched position between the robot-moved arm and the participant-moved arm, (2) Variability, which measures the trial-to-trial variance in the participant’s ability to match limb position, (3) Contraction Expansion, which describes the perception of the workspace in which the robot moved their affected arm as either shrunken or enlarged, and (4) Shift, which captures a perceived systematic shift of the robot moved arm. Each parameter was calculated in the x and y directions. Further details on these parameters and how they are calculated have been previously published [3,7,32].

Each parameter was first converted into a z-score based on a large normative data set, composed of 2229 previously collected APM task assessments from 799 control participants with no history of neurological disorders. For each parameter, data from the control set was first converted to a normal distribution by Box-Cox transformation and outliers were removed from the data that were outside ±3.29 SDs from the mean. Weighted linear regression models were used to remove the influence of age, sex and handedness [32,33,34]. Parameter z-scores were then calculated, with a z-score of zero equal to the mean performance of controls. 

The next step was to convert these parameter z-scores into the APM Task Score. Parameter z-scores were first transformed such that for any given parameter, the best score was indicated by a score of zero and larger values indicative of worse performance [32,33,34]. Next, the root sum square (RSS) distance was calculated for each of these transformed scores and converted into a z-score by the same transformations used to convert parameter scores to z-scores. The final step in calculating the APM Task Score was to convert the z-score of the RSS distance into a zeta-score using the zeta-transformation [32]. Robotic analysis was performed using Dexterit-E version 3.9 (Kinarm, Kingston, ON, Canada).

The APM Task Score allows for comparisons in performance between stroke and control participants to be made, accounting for each participant’s age, sex and handedness [32]. Since the APM Task Score is a normative score, based on a large control dataset (*n* = 799), it adopts the same features as a normal distribution. As such, 95% of healthy control participants, of the same age, sex and handedness as any given participant, have an APM Task Score less than 1.96 [32]. APM Task Scores greater than 1.96 indicated abnormal performance on the APM task and was used to infer if a participant had a proprioceptive impairment at six months. While the APM Task Score was used to quantify the binary proprioceptive outcome at six months, each of the parameter scores collected at two weeks were used as predictive features in the models trained. Further details on the APM Task Score, and each of these processes and calculations, have been extensively published and are best described in [34]. Additional documentation outlining these details can be freely downloaded at (https://kinarm.com/download/kst-summary-analysis-version-3-9/; accessed on 4 June 2022).

### 2.4. Clinical Assessment

Participants also completed a battery of clinical assessments two weeks post-stroke, collected along with the initial robotic assessment. Clinical assessments included the: Thumb Localization Test (TLT) [35], BIT [36] and FIM [37]. Scores from these clinical assessments were also used as features to predict proprioceptive impairments on the APM task at six months post-stroke, as described in Section 2.6.

The TLT was collected as a clinical measure of proprioception. In the TLT, with the participant’s eyes closed, the clinician moves the affected arm to a fixed position and asks the participant to try to pinch the thumb of that limb with the opposing limb. The clinician gives a score ranging from zero to three (0 = quickly and accurately locates thumb, 1 = locates thumb with a minor corrective movement, 2 = locates thumb by chance or uses hand or other fingers as a guide, and 3 = unable to locate thumb at all, or uses arm as a guide). The BIT was collected due to the close association between hemispatial neglect and proprioceptive impairment [15,16,17,18]. The conventional sub-tests of the BIT were used, which included the following pencil and paper tasks: line bisection, line cancellation, letter cancellation, star cancellation, shape copying, and figure drawing. Lower scores indicate worse attentional deficits, with scores less than 130 indicative of hemispatial neglect. It has been previously established that proprioceptive impairments are linked with reduced participation in ADLs [5,6,7]. As such, the FIM was collected as a measure of ADLs and indicative of overall stroke severity. The FIM measures the performance of ADLs across 18 items, including measures of self-care, locomotion, communication, and social cognition. 

### 2.5. Neuroimaging

For all participants, clinical MRI or CT imaging was collected in accordance with the acute stroke imaging procedure at the Foothill Medical Centre, Calgary, Alberta, Canada. The mean time from stroke to image acquisition was 2.9 ± 4.3 days. MRI images were collected on a 1.5 T or 3 T General Electric scanner. Acquisition sequences included fluid-attenuated inversion recovery (FLAIR) and diffusion-weighted imaging (DWI). CT images were collected on either a Siemens system or General Electric system. 

Participants’ lesions were marked on the original FLAIR or CT image, and the marking was verified by a stroke neurologist. The marked lesions were then normalized into Montreal Neurological Institute (MNI) space using the clinical toolbox (https://www.nitrc.org/projects/clinicaltbx; accessed on 13 January 2022) [38] in SPM12 (https://www.fil.ion.ucl.ac.uk/spm/software/spm12/; accessed on 13 January 2022). The normalized lesions were checked for accuracy by ensuring the alignment of the ventricles, anterior and posterior commissures and overall brain outline. Normalized lesions were then used to generate two neuroimaging measures, pertaining to each specific lesion, that were subsequently used as features in the prediction models. 

#### Neuroimaging Measures

The first neuroimaging measure used was a simple calculation of lesion volume for each participant’s lesion. A second neuroimaging measure was derived from Voxel-Based Lesion Symptom Mapping (VLSM) methodology [39]. Participants lesions and APM Task Scores, at two weeks post-stroke, were subject to an initial VLSM analysis. The VLSM analysis was performed using the NiiStat Toolbox (https://www.nitrc.org/projects/niistat; accessed 13 January 2019) in Matlab 2020a (Mathworks, Natick, MA, USA). At each voxel, participants were separated into those with and without lesions at that voxel. The voxel-wise significance was then determined for the difference in APM Task Scores between those with and without lesions to that voxel. To ensure statistical power, only voxels with minimum overlap of 5% (seven participants) were tested, which is an accepted threshold in the VLSM literature [20,40,41]. The result is a map where each voxel was assigned to a z-score from the corresponding statistical test. Next, to determine the structure-function relationship with respect to the APM Task Score, the mean z-score associated with all the voxels of each participant’s lesion was calculated (VLSM mean Z). Therefore, for each participant, the VLSM mean Z score is a scalar metric, weighted to the relative importance of all the lesioned voxels to the APM Task Score. 

### 2.6. Statistical Analysis

The purpose of this study was to assess the utility of clinical, neuroimaging and robotic measures, collected two weeks post-stroke, in predicting proprioceptive impairment on a robotic APM task six months post-stroke. 

To first validate the linear relationships between each clinical, neuroimaging and robotic measure collected at two weeks and six-month APM Task Scores, simple linear regressions were conducted. For the TLT, Spearman’s rank correlation coefficient was performed. Additionally, the relationship between demographic information (age, sex, affected side) was also assessed. For age, linear regression was also performed, whereas two sample t-tests were adopted for sex and affected side, assessing for differences in six-month APM Task Scores between males and females and left and right affected participants.

Next, participants were split into two groups, those impaired on the APM Task at six months (APM Task Score < 1.96) and those unimpaired on the APM task (APM Task Score > 1.96). For each clinical, robotic and neuroimaging measure, separate Mann–Whitney-U tests were conducted to test if group-level differences existed for each measure, between the impaired and unimpaired groups. To correct for multiple comparisons, a Bonferroni adjusted critical alpha of 0.00357 (14 comparisons) was used to infer significance. 

Finally, to assess the utility of clinical, neuroimaging and robotic measures for predicting six-month proprioceptive outcomes (impaired vs. unimpaired), logistic regression classifiers were trained. Firstly, a *Basic model* containing demographic information was trained, which formed the basis for each subsequent model. Then, to assess the predictive utility of each individual modality, models containing only clinical, neuroimaging or robotic features were trained (*Clinical model*, *Imaging model*, *Robotic model*). All the features were also combined into an *Augmented model*, to assess whether the addition of specialized robotic measures would improve the prediction of impairments over current clinically available information such as clinical assessments and neuroimaging. Within the *Augmented model*, coefficients from the logistic regression were used to determine the relative importance of each feature towards aiding the prediction. The features included in each model are presented in Table 1. Classification models were trained using the Scikit toolbox (version 1.1.3) in Python (version 3.9.13) and were cross validated using stratified 10-fold cross validation. 

The performance of each classification model was evaluated by calculating the classification accuracy, F1-score (to account for imbalances between the number of participants with and without impairments), area under the receiver-operator characteristic (ROC) curve (AUC), sensitivity, and specificity. It was anticipated that the *Augmented model* would yield more accurate predictions and reduce prediction error. To evaluate whether the potential benefits in prediction accuracy outweighed the cost of including additional features, Akaike information criterion (AIC) was also used to compare models, penalizing models with more features. 

## 3. Results

### 3.1. Participant Demographics and Other Predictors

**Figure 2:**
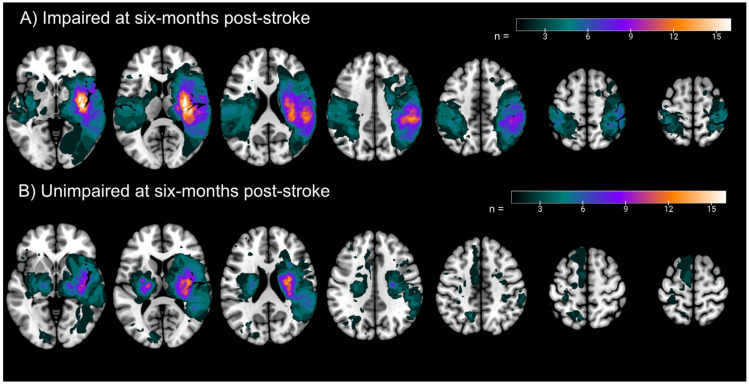
Lesion Overlaps—Lesion overlaps for (**A**) Participants impaired on the APM Task at six months post-stroke, and (**B**) Participants unimpaired on the APM Task at six months post-stroke. All lesions are normalised to Montreal Neurological Institute space and are presented in neurological convention (right hemisphere presented on the right). Color bar indicates the number of participants with lesions at each voxel. Axial slices (from left to right), z = −2, 10, 22, 34, 46, 58, 64.

A total of 133 participants were included in the current study (females = 42; Left affected arm = 78). Participant demographics are presented in Table 2. Participants were 60.2 ± 13.0 years of age. By six months post-stroke, 48 participants still had impairments on the APM task (36.1%). As such, a classification model that predicted every participant as impaired or unimpaired at six months (i.e., a single class/chance model), would have been 36.1% or 63.9% accurate, respectively. Lesion overlaps for participants impaired and unimpaired at six months are presented in Figure 2. At two weeks post-stroke, 26 participants had neglect (BIT scores < 130); however, only four participants still had neglect by six months, alleviating concerns that impairments on the APM task at six months could be due to participants still having neglect (Supplemental Figure S1).

### 3.2. Examining the Linear Relationships between Clinical, Neuroimaging and Robotic Features and Six-Month APM Task Scores

First, the linear relationships between each feature collected at two weeks post-stroke and six-month APM Task Scores were independently validated (Supplemental Figures S2–S4). All clinical features collected two weeks post-stroke were significantly associated with six-month APM Task Scores (Supplemental Figure S2). For the TLT (rho = 0.499, *p* = 9.92 × 10^−10^), higher scores (worse performance) were associated with higher APM Task Scores (worse performance). For the BIT (R^2^ = 0.253, *p* = 4.12 × 10^−10^) and FIM (R^2^ = 0.239, *p* = 1.39 × 10^−9^), lower clinical scores (worse performance) were associated with higher APM Task Scores (worse performance). Additionally, both neuroimaging features were significantly associated with six-month APM Task Scores (Supplemental Figure S2). Greater lesion volumes (R^2^ = 0.169, *p* = 5.29 × 10^−7^) and VLSM mean Z scores (R^2^ = 0.205, *p* = 2.78 × 10^−8^) were also all significantly associated with higher APM Task Scores (worse performance). Of the robotic features collected two weeks post-stroke, significant associations with six-month APM Task Scores were observed for Absolute Error X (R^2^ = 0.300, *p* = 5.46 × 10^−12^), Absolute Error Y (R^2^ = 0.447, *p* = 9.21 × 10^−19^),Variability X (R^2^ = 0.325, *p* = 4.64 × 10^−13^), Variability Y (R^2^ = 0.423, *p* = 1.49 × 10^−17^), Contraction Expansion X (R^2^ = 0.313, *p* = 1.58 × 10^−12^) and Contraction Expansion Y (R^2^ = 0.382, *p* = 1.44 × 10^−15^) (Supplemental Figure S3). There were no significant associations between two-week Shift X and Shift Y scores and six-month APM Task Scores.

For the demographic features, six-month APM Task Scores were significantly greater in left affected individuals than right affected individuals (t = 3.284, *p* = 0.0013) (Supplemental Figure S4). There were no differences between males and females (t = 1.815, *p* = 0.0718), nor a significant relationship with age (R^2^ = −0.00596, *p* = 0.641) (Supplemental Figure S4).

### 3.3. Examining Differences in Clinical, Robotic and Neuroimaging Features between Those Impaired and Unimpaired on the APM Task Score

**Figure 3:**
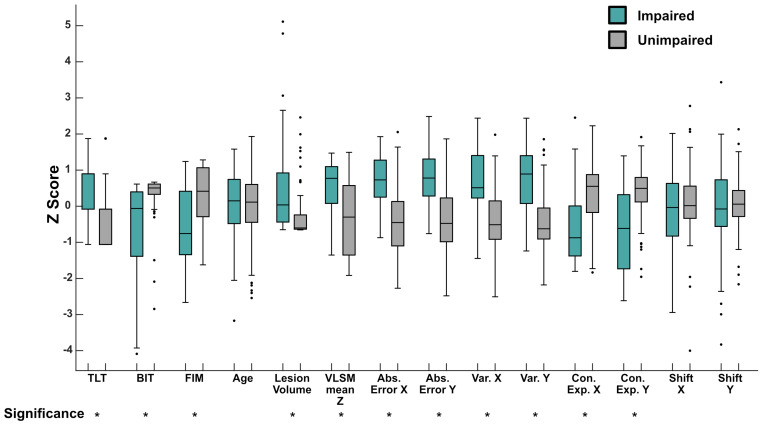
Group Level Differences in Clinical, Neuroimaging and Robotic Featuress Between Those Impaired and Unimpaired on the Arm Position Matching Task—Boxplots presenting features collected at two weeks post-stroke, for those impaired on the APM task (green) and those unimpaired (grey) at six months post-stroke. Given the varied scales each feature is scored on, scores are presented as z-scores for illustrative purposes. Boxes represent the median (centre line), 25th percentile (bottom line) and 75th percentile (top line), respectively. Whiskers extend to the highest and lowest data points, within 1.5 times the interquartile range from the top or bottom of the box. Individual data points displayed outside of this range signify outliers. Mann–Whitney U tests were conducted on the raw scores for each measure, between groups. * below each label indicates that feature had a significant difference between the impaired and unimpaired groups (*p* < 0.0005). Abs. Error = Absolute Error, Var = Variability, Con. Exp = Contraction Expansion.

The second analysis assessed whether there were differences in clinical, neuroimaging and robotic features, collected two weeks post-stroke, between participants with and without APM task impairments (APM Task Scores > 1.96) at six months post-stroke. With the exception of age (*p* = 0.974), Shift X (*p* = 0.294) and Shift Y (*p* = 0.944), significant differences were observed for all measures (all *p*-values < 0.0005) between those with and without impairments on the APM task (Figure 3). For those with impairments, all measures were significantly higher (worse performance), except for BIT, FIM and Contraction Expansion X and Y, which were significantly lower (worse performance—in the case of Contraction Expansion, lower values indicate more contraction).

### 3.4. Classification Models

#### 3.4.1. Single Modality Models

**Figure 4:**
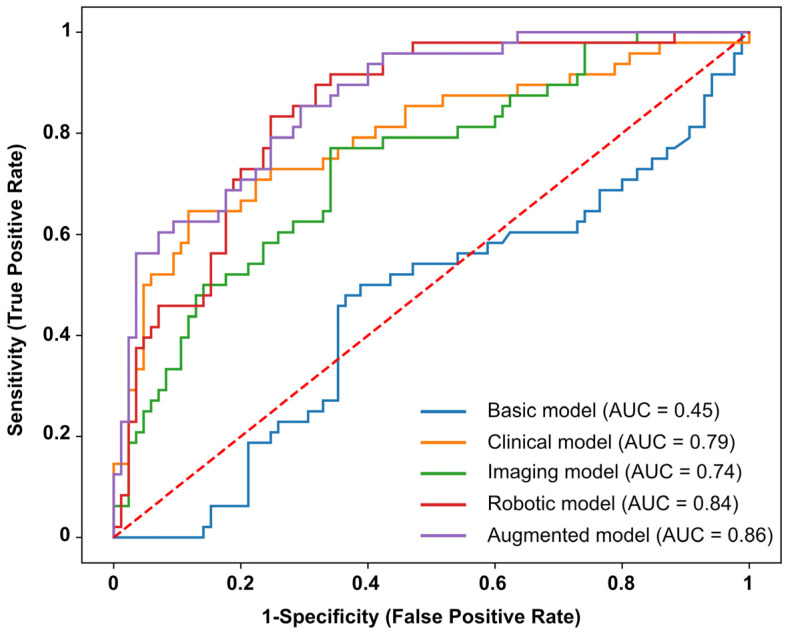
Receiver Operator Characteristic Curves (ROC curves)—Receiver Operator Characteristic (ROC) curves for the models predicting APM Task impairments at six months post-stroke. Each line represents the ROC curve for an individual model. Blue line = Basic model, orange line = Clinical model, green line = Imaging model, red line = Robotic model, purple line = Augmented model. Area under each curve (AUC) is reported in the figure legend. Dashed red line represents an AUC of 0.5 and a model that performs at random.

Performance metrics of each classification model are presented in Table 3, with ROC curves for each model presented in Figure 4. When classifying impairments at six months post-stroke, using data collected two weeks post-stroke, all models containing single modality features (e.g., *Clinical, Imaging* and *Robotic models*) performed reasonably well (Table 3), with the exception of the *Basic model*. The most accurate model was the *Clinical model* (Accuracy = 78.95%, F1-score = 0.78), closely followed by the *Robotic model* (Accuracy = 77.44%, F1-score = 0.77) and then the *Imaging model* (Accuracy = 72.18%, F1-score = 0.71). With regard to the AUC metric, the highest performing single modality model was the *Robotic model* (AUC = 0.84), followed by the *Clinical model* (AUC = 0.79) and then the *Imaging model* (AUC = 0.74). The *Robotic model* also had the lowest AIC value (AIC = 120.07), compared with the *Clinical model* (AIC = 146.96) and *Imaging model* (148.43). The *Basic model* performed poorly on all metrics, except for specificity (Table 3). The highest contributing features for each model are presented in Supplemental Table S1. For the *Clinical model*, TLT scores (0.9513) and Affected arm (−0.6551) were the most important features of the model. For the *Imaging model*, Affected Arm (−0.7606) and VLSM mean Z (0.6665) were most important. For the *Robotic model*, the prediction was mostly driven by Affected Arm (−0.9762), Variability Y (0.8015) and Absolute Error X (0.4643).

#### 3.4.2. Augmented Model

**Figure 5:**
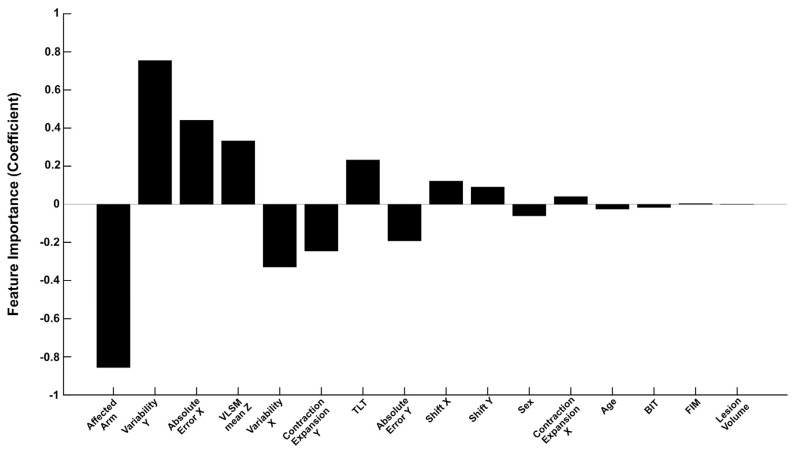
Augmented Model Feature Importance—Feature coefficients presented for the Augmented model. Features are ranked in order of absolute coefficient value. TLT = Thumb Localisation Task, BIT = Behavioural Inattention Test, FIM = Functional Independence Measure, VLSM = Voxel-based Lesion Symptom Mapping.

When combining all demographic, clinical, neuroimaging and robotic features, the *Augmented model* also performed well, with an accuracy of 76.69%, F1-score of 0.77 and an AUC of 0.86. Of all the models trained, the *Augmented model* had the highest AUC (Table 3). With the exception of the *Robotic model*, the *Augmented model* also had a lower AIC than all other models. Upon closer inspection of the model coefficients (Figure 5, Supplemental Table S1), there were five features, in particular, that contributed the most towards the *Augmented model* predictions. The largest contributors to the model prediction were: Affected Arm (−0.857), Variability Y (0.755), Absolute Error X (0.441), VLSM mean Z (0.333) and Variability X (−0.330) (Figure 5, Supplemental Table S1). Contraction Expansion Y (−0.245) and TLT scores (0.233) were also relatively important features.

## 4. Discussion

The idea of predicting functional outcomes after a stroke is not new, as many studies have focused on predicting motor recovery and ADLs post-stroke [42,43,44,45,46,47]. Much less attention, however, has been placed on predicting sensory and proprioceptive recovery. This study demonstrated the utility of clinical, neuroimaging, and robotic measures, collected two weeks post-stroke, for predicting proprioceptive outcomes at six months post-stroke. Models which only contained a single modality of features (e.g., only clinical, neuroimaging or robotic features) resulted in prediction accuracies for long-term impairment that were greater than a model performing at chance, ranging from 72 to 79% (Table 3). Surprisingly, however, the combination of robotic features with clinical and neuroimaging features did not improve prediction accuracy or F1-score over the single modality models. When evaluating model performance based on AUC and AIC, there was, however, a clear advantage for models that utilized robotic features over those without (Table 3, Figure 4). The higher AUC, along with a relatively low AIC value, validates the use of models that are rich in features derived from robotic assessments when predicting long-term proprioceptive outcomes. Overall, this study advances our understanding of the predictors of proprioceptive recovery, something which has recently been called for in stroke recovery research [29]. Developing this understanding is important for identifying individuals who are at risk of long-term impairment, who could benefit from additional proprioceptive rehabilitation in the first six months post-stroke.

Independent of clinical and neuroimaging features, the use of robotic features resulted in a prediction accuracy of 77.44%. Although the accuracy and F1-score of the models containing robotic features were similar to those containing just clinical or neuroimaging features, the *Robotic* and *Augmented models* outperformed the *Clinical* and *Imaging models* in terms of AUC and AIC metrics. The relatively higher AUC suggests a greater ability of the models containing robotic features to separate those with proprioceptive impairments at six months from those without. Additionally, the lower AIC supports the use of the extra robotic features in these models. The improvements observed in AUC but not in accuracy are likely a reflection of what each metric measures. While accuracy is simply the proportion of correct predictions at a single model threshold, AUC measures the relationship between the True Positive Rate (Sensitivity) and the False Positive Rate (1-Specificity) at different threshold values. AUC is also biased towards the positive class (in this case, the impaired class). As seen in Figure 4, the *Robotic* and *Augmented model’s* performance separates from the *Clinical* and *Imaging models* as the True Positive Rate exceeds 0.7 (i.e., a higher True Positive Rate/Sensitivity for a lower False Positive Rate), suggesting the greater ability for the models containing robotic features to correctly identify the impaired participants across a wide range of threshold values.

To date, the literature is limited when predicting and classifying sensory outcomes after stroke. Other classification studies have, however, attempted to predict functional outcomes post-stroke [42,43,45,46,48] based on the Modified Rankin Scale or Barthel Index. Many of these have been deemed successful, reporting accuracies in the range of 56–85% [43,45] and AUC values in the range of 0.76–0.91 [42,48,49,50]. The *Clinical*, *Robotic* and *Augmented models* in the current study had comparable accuracies and AUC values, ranging between 76.69–79.95% and 0.79–0.86, respectively. Furthermore, in contrast to proprioception, there is a far deeper body of the literature aimed at predicting upper limb motor recovery, with a particular focus on clinical and physical markers such as finger extension and shoulder abduction, neurophysiological markers such as the presence of motor evoked potentials and neuroimaging markers such as corticospinal tract integrity [51,52,53] (for a comprehensive review of the literature, see [54]). That said, similarities are shared with those in the motor literature, whereby clinical and neuroimaging features contributed to successful prediction of proprioceptive outcomes in this study. In order to fully critique the performance of the models trained in this study, further research is required that attempts to predict sensory outcomes post-stroke. Doing so would allow the effectiveness of the models trained in this study to be compared with other suitable literature. As the body of literature grows surrounding sensory outcome prediction post-stroke, more tailored models can be trained that utilize a growing wealth of knowledge.

The present study has strong implications for stroke rehabilitation and promoting a more routine use of, and investments in, robotic technology in clinical practice. Being able to accurately assess proprioception and predict the likelihood of long-term impairments is an integral step towards developing better treatment plans for patients identified at risk of long-term impairments, who would otherwise have reduced independence in daily living and quality of life [7,55]. Robotics may be an important tool in improving both sensory prediction and assessment post-stroke.

Importantly, within the *Robotic* and *Augmented models*, this work has identified key components from a robotic assessment of proprioception that, when collected early post-stroke, are indicative of long-term proprioceptive problems for patients. In addition to the successful performance of the *Robotic model*, there were key features of the *Augmented model*, derived from robotic assessments, that were major contributors to predicting outcomes, for example, Variability Y, Absolute Error X and Variability X (Figure 5, Supplemental Table S1). This is of particular importance as rehabilitation looks towards a personalized medicine approach. Since robotics can identify key areas that predict long-term impairments, they have the capacity to inform the rehabilitation process by aiding the design of targeted interventions for these specific areas and mitigating long-term impairments. Robotics are advantageous for assessing proprioception as they provide an abundance of information about someone’s performance and are far more precise than clinical assessments [31], collecting data every millisecond (in the case of the Kinarm Exoskeleton used in this study). As such, robotics are optimally suited to provide additional details about a person’s particular proprioceptive impairment. Additionally, compared to the clinical assessments performed in this study (TLT, BIT and FIM), which can take up to 30 min to perform and require a trained clinician/rater, the APM task provides plentiful data in under four minutes of assessment time. Taken together, the present findings support the use of robotics to inform and improve the prediction of long-term patient outcomes, as well as inform rehabilitation.

Despite limited clinical uptake for the treatment of post-stroke proprioception, some potentially promising evidence comes from experimental interventions [12]. These studies, which are limited and only conducted in small samples, have been shown to increase proprioceptive acuity following stroke using a variety of treatment methods [56,57,58,59,60,61,62,63,64,65]. Many of these studies have utilized alternative treatments such as robotic therapy [57,59,60,63] including robotic mirror therapy [65] and assisted movement with enhanced sensation [58] but have also included more traditional tactile and proprioceptive discrimination training [56]. Interestingly, robotic therapy has also been proven to be beneficial for treating motor function after a stroke [66,67]. Given the capability of robotics to facilitate prediction of proprioceptive impairment, either alone or in conjunction with clinical assessments, the use of robotics in clinical settings in addition to their apparent benefit for proprioceptive rehabilitation should continue to be explored and considered.

It has previously been demonstrated that the ability to predict functional outcomes post-stroke, using clinical measures alone, hits a ceiling of prediction accuracy at around 80% [43,45], with the addition of specialized features (such as neuroimaging or robotics) potentially required to facilitate improvements in model performance [43]. Although it was anticipated that robotic features would result in higher prediction accuracy than clinical or neuroimaging features, unfortunately this was not found to be the case, with the *Robotic* and *Augmented models* performing similarly to the *Clinical and Imaging models*.

It must be noted, however, that the *Clinical model* still performed well, even in the absence of more specialized features such as neuroimaging or robotics. Within this model, TLT scores and the affected arm were the most informative features (Supplemental Table S1). The TLT was also amongst the more informative features of the *Augmented model* (Figure 5, Supplemental Table S1). The relative success of using clinical measures for long-term prediction corroborates the findings of Reid et al. [42], who demonstrated that outcome predictions, based on the Modified Rankin Scale, were just as good using simple clinical measures compared to more complex models which utilized neuroimaging measures. The ability of the clinical measures used in the current study to predict proprioceptive outcome also may not be surprising, considering the close associations that have previously been described between attentional deficits, the ability to perform ADLs and proprioception post-stroke [7,15,18]. The high prediction accuracy in this study may therefore reflect these tight relationships between clinical and robotic measures of proprioception (Supplemental Figure S2). While robotics provide distinct advantages over clinical assessments [31], they are not common in clinical sites. In sites where robotics are not accessible, it is important that clinicians recognize the associations between these types of clinical measures and the likelihood of long-term proprioceptive difficulties in their patients, when performing assessments early after stroke and prescribing rehabilitation.

Finally, within the *Augmented model*, the affected arm and VLSM mean Z also contributed to the model prediction (Figure 5, Supplemental Table S1). There is a growing body of evidence that suggests that proprioception is lateralized to the right hemisphere, with a distributed network of brain regions likely responsible for proprioception [16,68,69,70,71,72,73,74,75,76]. With APM Task Scores greater (worse) in left-arm affected individuals (Supplemental Figure S4) and greater VLSM mean Z scores in those with impairments at six months, the relative importance of these measures as predictors of long-term impairment likely reflects this lateralized neuroanatomy for proprioception.

### Limitations

Like all studies, the current study has limitations. Given the size of the dataset, models were both trained and tested within samples and the test data were not necessarily unseen. While it is suggested that using the same data to train and test models might be useful from the standpoint of exploring potential predictive measures [77,78,79], in an ideal scenario a vastly larger dataset would be available, allowing these findings to be generalized to a wider sample. In this study stratified 10-fold cross-validation was used, which can protect against these biases. It must also be noted that there were slight imbalances in the number of participants with and without impairments in the sample (approximately 60–40% split), something which was maintained through each cross-validation sample. This imbalance may have the potential to bias the current findings and distort the perceived performance of each model trained. That said, all the models outperformed a single class model by 10–15% accuracy.

## 5. Conclusions

Predicting proprioceptive impairments out to six months post-stroke using clinical, neuroimaging and robotic measures, collected within the first two weeks post-stroke, is feasible. Considering the current clinical standard of care often does not focus on treating proprioceptive impairments, identifying those likely to have long-lasting impairments is a key step towards personalized approaches in the treatment of proprioception. Further studies are, however, needed to refine predictive models and increase the prediction accuracy of proprioceptive recovery post-stroke.

## Figures and Tables

**Table 1:** Model variables—Features entered into each predictive model. TLT = Thumb Localisation Task, BIT = Behavioural Inattention Test, FIM = Functional Independence Measure, VLSM = Voxel-based Lesion Symptom Mapping.

Model	Measures Included
Basic model	Age, Sex, Affected Arm
Clinical model	Age, Sex, Affected Arm, TLT, BIT, FIM
Imaging model	Age, Sex, Affected Arm, VLSM mean Z, Lesion Volume
Robotic model	Age, Sex, Affected Arm, Absolute Error X, Absolute Error Y, Variability X, Variability Y, Contraction Expansion X, Contraction Expansion Y, Shift X, Shift Y
Augmented model	Age, Sex, Affected Arm, TLT, BIT, FIM, VLSM mean Z, Lesion Volume, Absolute Error X, Absolute Error Y, Variability X, Variability Y, Contraction Expansion X, Contraction Expansion Y, Shift X, Shift Y

**Table 2:** Demographics—Participant demographics. For Age, Lesion Volume, VLSM mean Z, Absolute Error, Variability, Contraction Expansion, Shift and six-month APM Task Score, values are presented as the mean ± standard deviation. For the BIT and FIM, values presented are the median with the range presented in parentheses. BIT = Behavioural Inattention Test, FIM = Functional Independence Measure, TLT = Thumb Localisation Task, APM = Arm Position Matching, VLSM = Voxel-based Lesion Symptom Mapping.

**Age**	60.2 ± 13.0
**Sex**	Males = 91, Females = 42
**Affected Arm**	Right = 55, Left = 78
**TLT**	0 = 50, 1 = 36, 2 = 33, 3 = 14
**BIT**	141 (58–146)
**FIM**	102 (35–126)
**Lesion Volume (cc)**	35.1 ± 53.5
**VLSM Mean Z**	1.652 ± 1.223
**Absolute Error X (z-score)**	1.42 ± 1.30
**Absolute Error Y (z-score)**	1.49 ± 1.28
**Variability X (z-score)**	2.15 ± 1.93
**Variability Y (z-score)**	2.67 ± 2.12
**Contraction Expansion X**	−1.71 ± 2.20
**Contraction Expansion Y**	−2.27 ± 3.77
**Shift X**	−0.40 ± 1.78
**Shift Y**	−0.64 ± 1.98
**APM Task Score (six months)**	1.90 ± 1.55

**Table 3:** Predictive model performance—Performance metrics for each of the predictive models trained. For accuracy, sensitivity and specificity, values presented are percentages. * indicates the model(s) with the best performance for each given metric. For all metrics, except AIC, higher values indicate better performance. AUC = Area under the Receiver-Operator Characteristic curve, AIC = Akaike Information Criterion.

	Accuracy	F1-Score	AUC	Sensitivity	Specificity	AIC
**Basic Model**	63.16	0.49	0.45	0.00	98.82 *	176.94
**Clinical model**	78.95 *	0.78 *	0.79	62.50	88.24	146.96
**Imaging model**	72.18	0.71	0.74	47.92	85.88	148.43
**Robotic model**	77.44	0.77	0.84	68.75*	82.35	120.07 *
**Augmented model**	76.69	0.77	0.86*	64.58	83.53	126.07

## Data Availability

The data presented in this study is not publicly available due to restrictions on data sharing without formal data sharing agreements in place, as determined by the Institutional Review Board. Those wishing to obtain the study data should contact the corresponding author (Sean Dukelow).

## Supplementary Materials

The following supporting information can be downloaded at: https://www.mdpi.com/article/10.3390/brainsci13060953/s1, Supplementary Figure S1: Relationship between six-month BIT scores and APM Task Scores; Supplementary Figure S2: Examining the relationships between two-week clinical and neuroimaging measures and six-month Arm Position Matching Task Scores; Supplementary Figure S3: Examining the relationships between two-week robotic measures and six-month Arm Position Matching Task Scores; Supplementary Figure S4: Examining the relationships between demographic measures and six-month Arm Position Matching Task Scores; Supplementary Table S1: Predictive model coefficients.
