# Supplementary material for: Clinical, Neuroimaging and Robotic Measures Predict Long-Term Proprioceptive Impairments following Stroke
Source: Brain Sci. 2023 Jun 15;13(6):953. doi: 10.3390/brainsci13060953 (PMC10296129; doi:10.3390/brainsci13060953)
Supplement: Supplementary file 1 [file brainsci-13-00953-s001.zip › brainsci-2424213-supplementary.pdf]

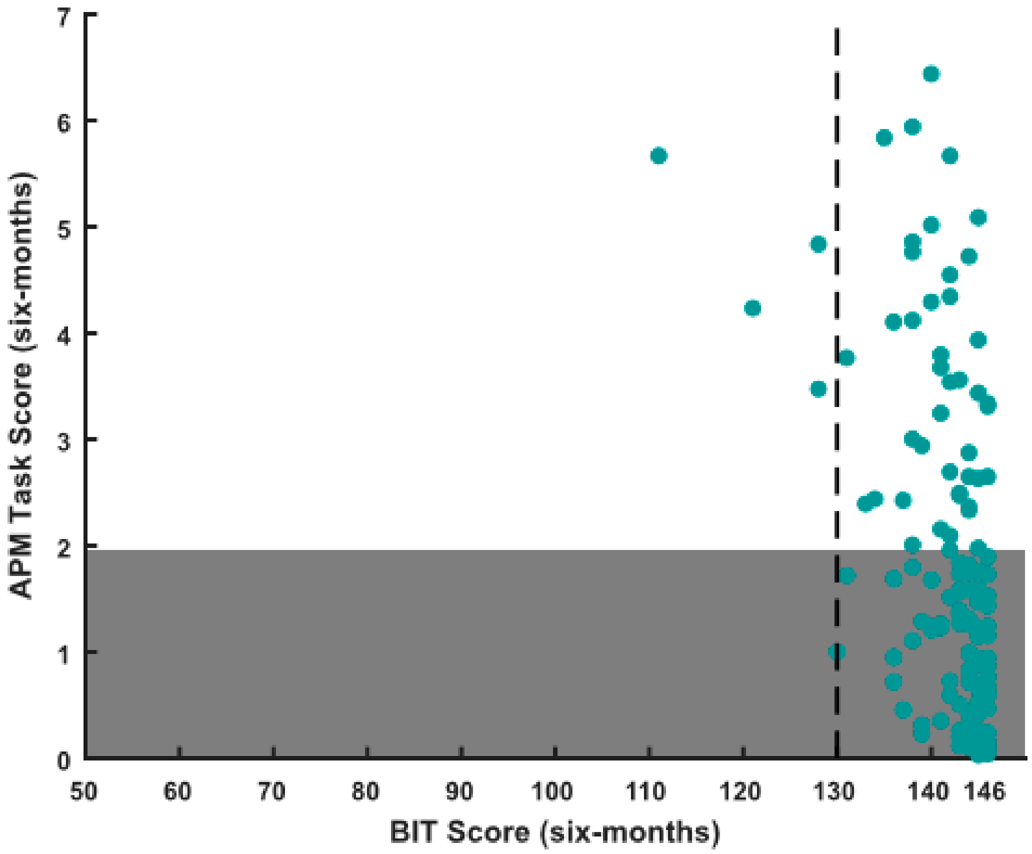

Supplemental Figure S1. Relationship between six-month BIT scores and APM Task Scores - Behavioural Inattention Test (BIT) scores, collected at six-months post-stroke and APM Task Scores. Grey area denotes the normal range of performance for healthy controls on the APM task. Data points outside of this range indicate participants with impairments on the APM task. Dashed line at a BIT score of 130, indicates the cut-off BIT score. Points to the left of this line (below 130) indicate the presence of hemispatial neglect six-months post-stroke. BIT = Behavioural Inattention Test.

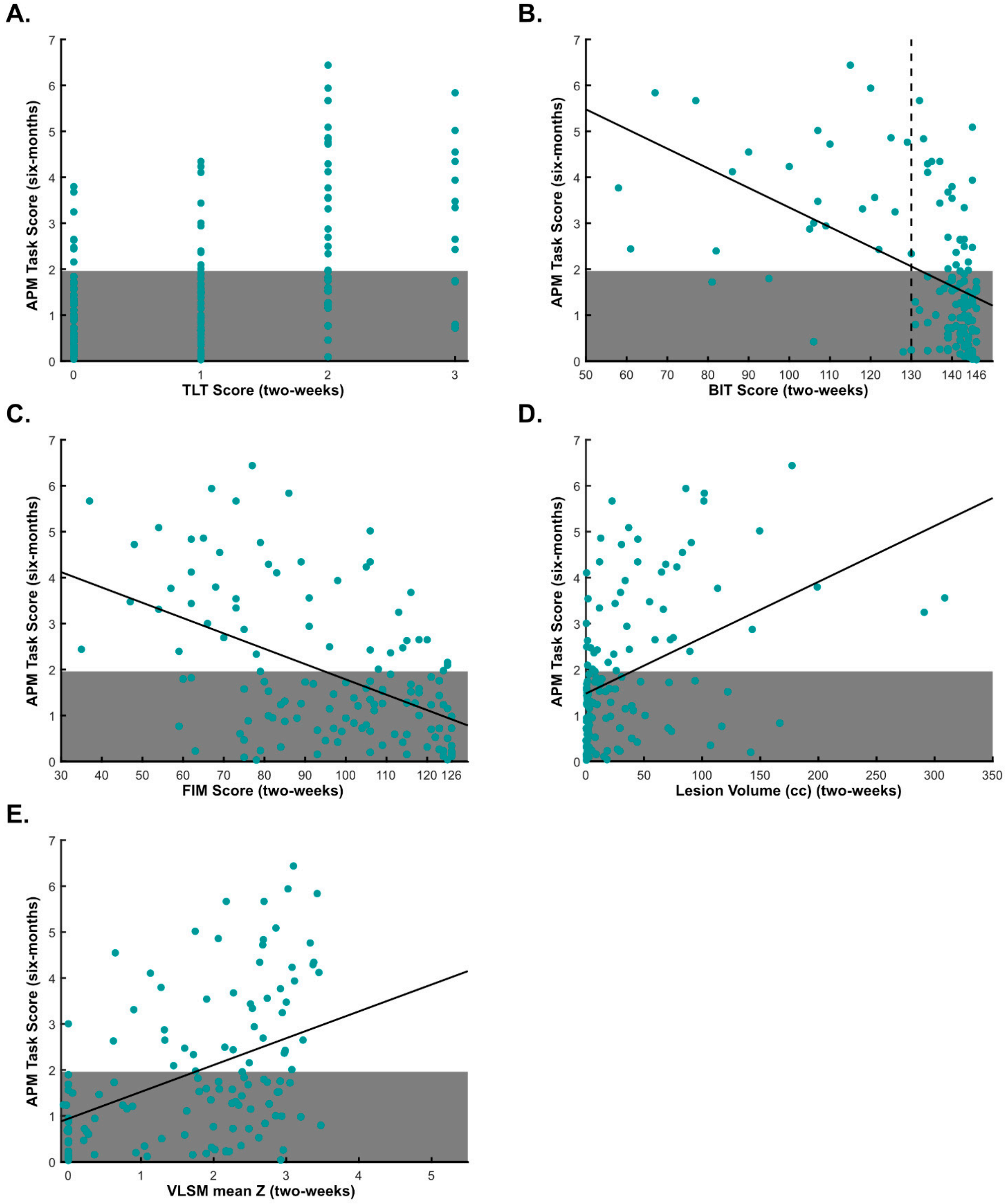

Supplemental Figure S2. Examining the relationships between two-week clinical and neuroimaging measures and six-month Arm Position Matching Task Scores – Plots display the relationship between clinical and neuroimaging measures collected at two-weeks post-stroke and Arm Position Matching Task Scores at six-months post-stroke. A) Thumb Localisation Test scores, B) Behavioural Inattention Test

scores, C) Functional Independence Measure scores, D) Lesion Volume and E) VLSM Mean Z scores. Individual data points are presented for each measure. Grey area denotes the normal range of performance for healthy controls on the APM task. Data points outside of this range indicate participants with impairments on the APM task. Dashed line in panel B, at a score of 130, indicates the cut-off BIT score. Points to the left of this line (below 130) indicate the presence of hemispatial neglect. TLT = Thumb Localisation Test, BIT = Behavioural Inattention Test, FIM = Functional Independence Measure, VLSM = Voxel-based Lesion Symptom Mapping.

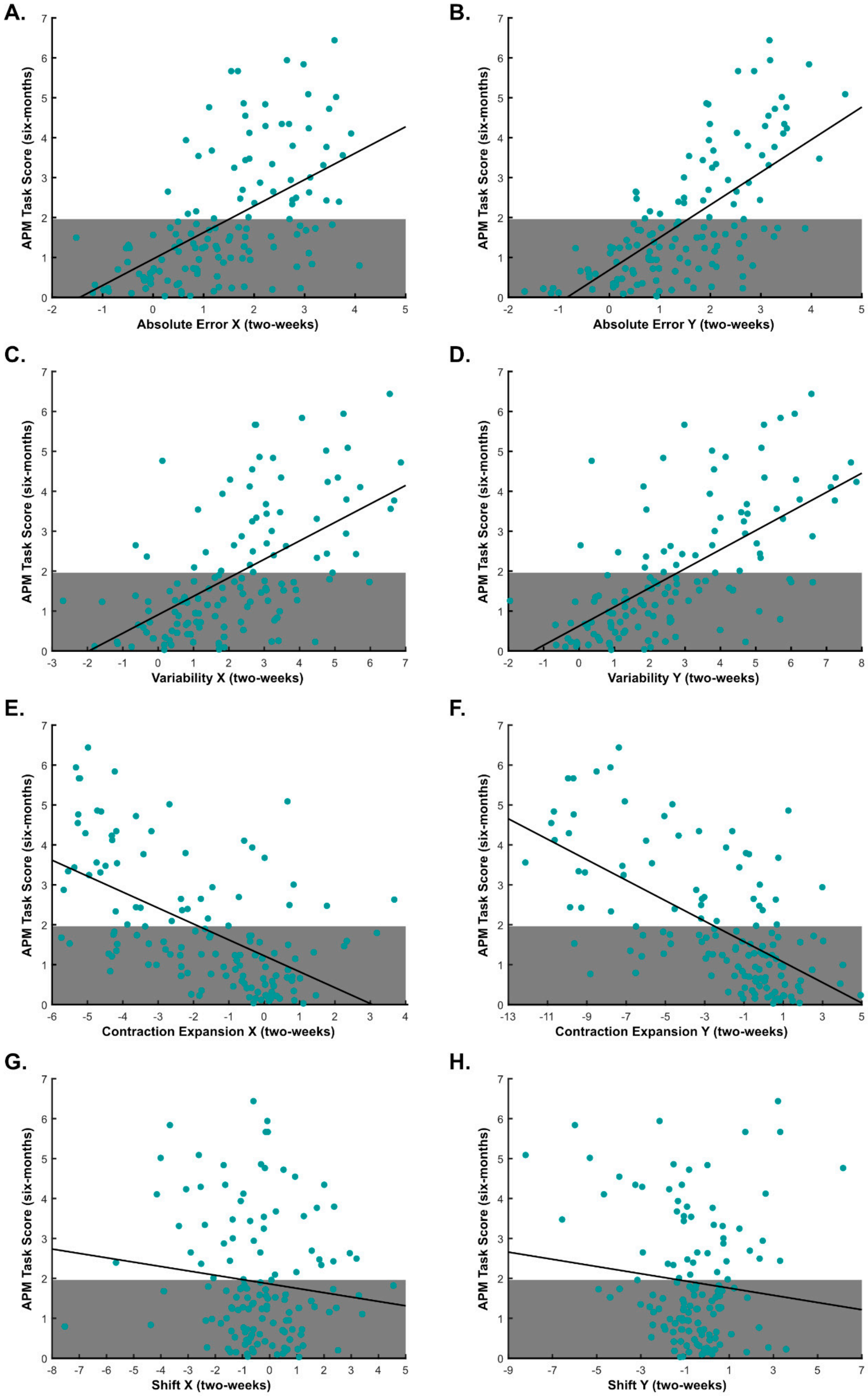

A.
APM Task Score (six-months)
Absolute Error X (two-weeks)
B.
APM Task Score (six-months)
Absolute Error Y (two-weeks)
C.
APM Task Score (six-months)
Variability X (two-weeks)
D.
APM Task Score (six-months)
Variability Y (two-weeks)
E.
APM Task Score (six-months)
Contraction Expansion X (two-weeks)
F.
APM Task Score (six-months)
Contraction Expansion Y (two-weeks)
G.
APM Task Score (six-months)
Shift X (two-weeks)
H.
APM Task Score (six-months)
Shift Y (two-weeks)

Supplemental Figure S3. Examining the relationships between two-week robotic measures and six-month Arm Position Matching Task Scores - Plots display the relationship between robotic measures collected at two-weeks post-stroke and Arm Position Matching Task Scores at six-months post-stroke. A) Absolute Error X, B) Absolute Error Y, C) Variability X, D) Variability Y, E) Contraction Expansion X, F) Contraction Expansion Y, G) Shift X, and H) Shift Y. All scores are parameter z scores. Individual data points are presented for each measure. Grey area denotes the normal range of performance for healthy controls on the APM task. Data points outside of this range indicate participants with impairments on the APM task.

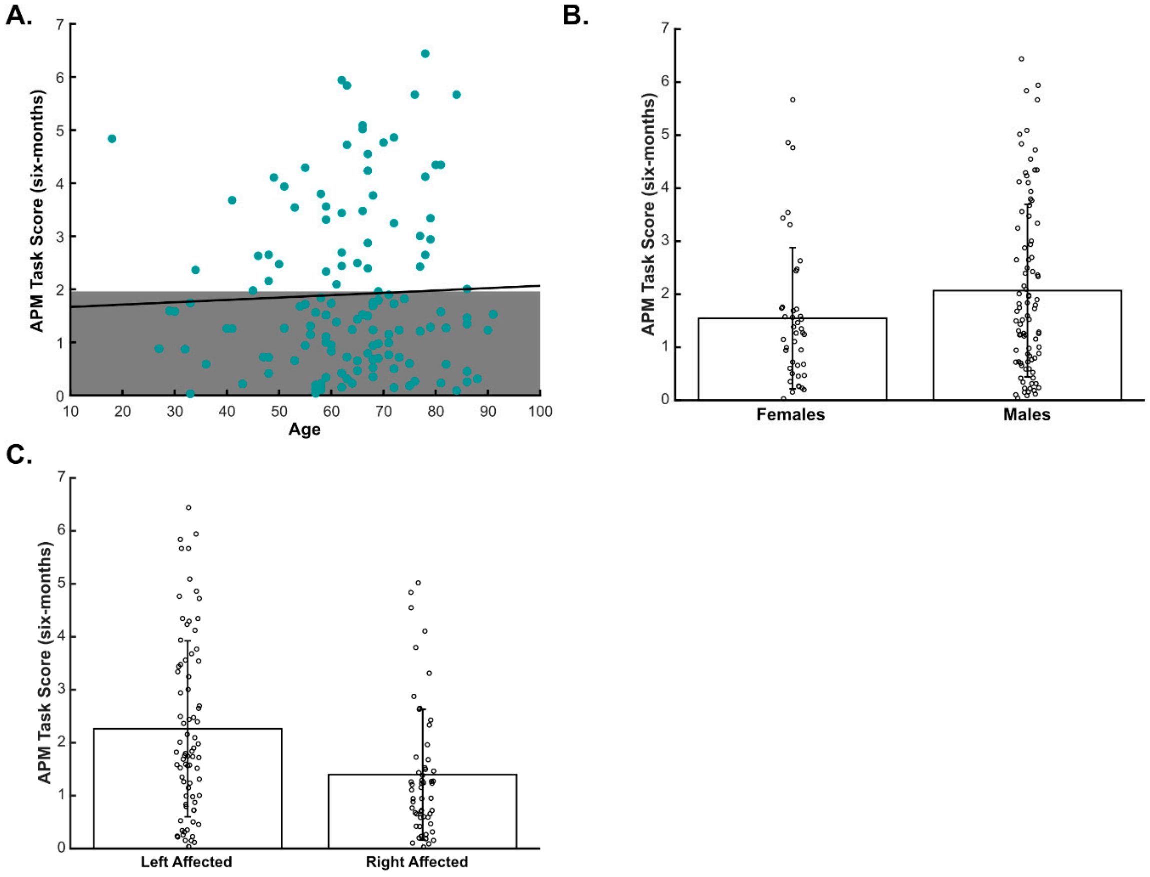

Supplemental Figure S4. Examining the relationships between demographic measures and six-month Arm Position Matching Task Scores – A) Plot displays the relationship between Age and six-month APM Task Scores. Grey area denotes the normal range of performance for healthy controls on the APM task. Data points outside of this range indicate participants with impairments on the APM task. B) and C) display six-month APM Task for females and males (B), and left and right affected individuals (C). Group means are presented, with standard deviations and individual data points also shown.

	Coefficient	Std. Error	p-value	CI [95%]
**Basic Model**				
Age	-0.0027	0.004	0.549	-0.011 0.006
Sex	-0.0708	0.389	0.856	-0.834 0.693
Affected Arm	-0.6633	0.357	0.063	-1.363 0.036
**Clinical Model**				
Age	0.0185	0.012	0.129	-0.005 0.042
Sex	0.2055	0.452	0.650	-0.681 1.092
Affected Arm	-0.6551	0.427	0.125	-1.493 0.182
TLT	0.9513	0.224	0.000	0.513 1.390
BIT	-0.0130	0.010	0.191	-0.032 0.006
FIM	-0.0100	0.011	0.365	-0.032 0.012
**Imaging Model**				
Age	-0.0269	0.007	0.000	-0.042 -0.012
Sex	-0.2184	0.457	0.632	-1.113 0.676
Affected Arm	-0.7606	0.406	0.061	-1.556 0.035
VLSM Mean Z	0.6665	0.198	0.001	0.278 1.054
Lesion Volume	1.242×10^{-5}	4.79×10^{-6}	0.009	3.04×10^{-6} 2.18×10^{-5}
**Robotic Model**				
Age	-0.0485	0.011	0.000	-0.071 -0.026
Sex	-0.2090	0.542	0.700	-1.271 0.853
Affected Arm	-0.9762	0.533	0.067	-2.022 0.069
Absolute Error X	0.4643	0.362	0.200	-0.245 1.174
Absolute Error Y	-0.0292	0.409	0.943	-0.830 0.772
Variability X	-0.2706	0.322	0.400	-0.901 0.360
Variability Y	0.8015	0.313	0.010	0.188 1.415
Contraction Expansion X	-0.0098	0.156	0.950	-0.316 0.296
Contraction Expansion Y	-0.2829	0.106	0.008	-0.491 -0.074
Shift X	0.0373	0.137	0.785	-0.231 0.305
ShiftY	0.1654	0.131	0.207	-0.091 0.422
**Augmented Model**				
Age	-0.0253	0.019	0.189	-0.063 0.012
Sex	-0.0606	0.588	0.918	-1.213 1.091
Affected Arm	-0.8566	0.570	0.133	-1.974 0.261
TLT	0.2328	0.341	0.495	-0.436 0.902
BIT	-0.0166	0.013	0.206	-0.042 0.009
FIM	0.0037	0.015	0.809	-0.026 0.033
VLSM Mean Z	0.3332	0.319	0.296	-0.292 0.958
Lesion Volume	2.291×10^{-6}	6.00×10^{-6}	0.703	-9.47×10^{-6} 1.41×10^{-5}
Absolute Error X	0.4409	0.369	0.233	-0.283 1.165
Absolute Error Y	-0.1918	0.454	0.672	-1.081 0.697
Variability X	-0.3296	0.338	0.329	-0.991 0.332
Variability Y	0.7546	0.320	0.018	0.127 1.382
Contraction Expansion X	0.0401	0.162	0.805	-0.278 0.358
Contraction Expansion Y	-0.2448	0.115	0.034	-0.471 -0.019
ShiftX	0.1218	0.147	0.407	-0.166 0.409
ShiftY	0.0902	0.145	0.534	-0.194 0.374

Supplemental Table S1. Predictive model coefficients – Coefficients, standard error, p values and 95% confidence intervals presented for each measure within each predictive model. Absolute coefficients used to infer the relative importance of a given feature to each model. TLT = Thumb Localisation Test, BIT = Behavioural Inattention Test, FIM = Functional Independence Measure, VLSM = Voxel-based Lesion Symptom Mapping.
